# Early detection breast cancer: role of circulating plasma miRNA-21 expression as a potential screening biomarker

**DOI:** 10.3906/sag-2005-138

**Published:** 2021-04-30

**Authors:** Muhammad Noor DIANSYAH, Ami Ashariati PRAYOGO, Made Putra SEDANA, Merlyna SAVITRI, Pradana Zaky ROMADHON, Putu Niken Ayu AMRITA, Andi Yasmin WIJAYA, Winona May HENDRATA, Ugroseno Yudho BINTORO

**Affiliations:** 1 Department of Internal Medicine, Dr. Soetomo Teaching Hospital, Surabaya Indonesia; 2 Department of Internal Medicine, Airlangga University Hospital, Surabaya Indonesia; 3 Faculty of Medicine, Airlangga University, Surabaya Indonesia

**Keywords:** Biomarkers, breast neoplasms, diagnosis, human plasma, miRNA-21

## Abstract

**Background/aim:**

To explore the potential of the circulating plasma miRNA-21 as an early detection biomarker by comparing earlystage breast cancer (BG) and healthy control (HG) in Indonesian population.

**Materials and methods:**

The enlisted patients were 26 adult female early-stage breast cancer patients (stage 1A, 1B, 2A, and 2B) of Airlangga University Hospital from August 2019 to October 2019. Sixteen volunteers were recruited as matching healthy subjects. MiRNA-21 expression was quantified by plasma qRT-PCR. Data analysis performed using IBM SPSS Statistics v.24 (IBM Corp., Armonk, NY, USA). MiRNA-21 cut-off, sensitivity, and specificity were analyzed using receiver operating characteristic (ROC) curve.

**Results:**

The study included 26 BG and 16 HG subjects. The miRNA-21 expression in BG group was 3.933 (1.181-11.794) and 0.905 (0.164-4.532) in HG group (4.34 folds; P = 0.001), with 1.66 cut-off (92.3% sensitivity; 81.2% specificity). MiRNA-21 expression separated analysis in HG showed a 0.578 times lower expression in menopause subjects [0.651 (0.164-0.414)], compared to premenopause ones [(1.123 (0.758 - 4.532); P = 0.031]. Yet, in BG group, 1.729 times higher miRNA-21 expression was observed in menopause subjects (6.021 ± 3.583), compared to premenopause ones (3.500 ± 1.517; P = 0.022).

**Conclusion:**

Circulating miRNA-21 expression is a potential biomarker for early detection of breast cancer and might act as a breast cancer risk predictor.

## 1. Introduction

Breast cancer is the malignancy of the breast tissues originating from uncontrolled proliferation of breast ductal and lobular epithelial cells[1]. Breast cancer is known as the second most prevalent malignancy in women worldwide [2]. The trend of breast cancer and overall cancer incidence has also showed an increase in developing countriesInternational Agency for Research on Cancer (2018). Population Fact Sheets: Indonesia [online]. Website https://gco.iarc.fr/today/data/factsheets/populations/360-indonesia-fact-sheets.pdf [accessed 18 February 2020].. Particularly in Indonesia, breast cancer is placed as the most prevalent cancer. In fact, more than 80% of breast cancer cases is diagnosed in advanced stageKementrian Kesehatan Republik Indonesia (2016). Panduan Penatalaksanaan Kanker Payudara (in Indonesian) [online]. Website http://kanker.kemkes.go.id/guidelines_read .php?id=2&cancer=1 [accessed 22 February 2020].. Triple test diagnostic approach (simultaneous physical examination, radiological examination, and histopathological examination) for determining the malignancy of breast tissues is an important procedure. However, this approach still has its limitations, especially in early detection of breast cancer [3]. Histopathology examination is the gold standard of breast cancer diagnostic, yet this examination is constrained by its invasiveness [4]. Radiology examination exposes the patient to radiation, with a disadvantage in the young patient [5], and the breast ultrasound examination could be operator dependent [6]. 

Besides the triple test and ultrasound examination, several blood tumor biomarkers are routinely used in medical practice with their specific roles. Tumor markers, such as carbohydrate antigen 15-3 (CA 15-3) and carcinoembryonic antigen (CEA), provide clinicians with information about cancer therapeutic response in advanced cancer stage and lack of practical information in early-stage breast cancer [7–9]. Besides, circulating blood tumor markers also showed relatively poor sensitivity and specificity [8,9]. 

MicroRNA (miRNA) is a small class noncoding RNA with 22-25 lengths of nucleotides that are present in the body fluid and tissues [10]. MiRNA plays an important role in gene expression as a critical gene regulator through epigenetic mechanism [10]. MiRNA acts as a silencer of messenger RNA (mRNA) through mRNA degradation, which impacts mRNA translation and expression. This mechanism affects the downstream cellular process, including cell maturation, cell differentiation, cell invasion, metastasis, and apoptosis [10]. Dysregulation of miRNA-related processes by mutation, deletion, translocation, and an increase or decrease of expression eventually could impair cell metabolism [11]. Several specific miRNAs suppress oncoproteins expression in normal cells. Nonetheless, increasing expression of different particular miRNA was also noticed in malignancy [11]. 

In breast cancer, there are several miRNAs, which tend to be upregulated (miRNA-21, miRNA-10b, miRNA-125b, miRNA-145, miRNA-155, and miRNA-191) and downregulated (miRNA-7, miRNA-17p, miRNA-27b, miRNA-143, dan miRNA-139) [12,13]. In particular, miRNA-21 is known to be characterized in breast cancer [10,14,15].

MiRNA-21 is an intron miRNA, coded in chromosome 17q23.1. MiRNA-21 expression affected cell proliferation, cell invasion, metastasis, and apoptosis [10]. MiRNA-21 target involves a group of tumor suppressor genes, including TPM1, TIMP3, RECK, p53 network, Cdc25A, PTEN, PDCD4, and Maspin [10]. MiRNA-21 could trigger breast cell proliferation and transformation by repressing the translation of tumor suppressor gene PDCD4, which is involved in apoptosis regulator [10,16]. 

MiRNA-21 expression could be detected in the tumor tissues, serum, plasma, and cerebrospinal fluid [4]. Expression of miRNA-21 in breast cancer tissues showed variability; however, miRNA-21 expression in blood circulation tends to be more stable [4,17]. 

High expression of circulating miRNA-21 might be an indicator of breast cancer [18]. Several studies evaluate the cut-off point of miRNA-21 resulting in variability of cut-off point, specificity, and sensitivity for miRNA-21 expression [8,19,20]. Despite the variability, studies of miRNA-21 expression overall show promising sensitivity and specificity for detecting breast cancer. Circulating miRNA-21 expression levels in Indonesian breast cancer patients have never been assessed before. Hence, it is needed to assess miRNA-21 potential as a breast cancer early biomarker in Indonesian population, by comparing circulating expression of miRNA-21 in early-stage breast cancer patients compared to a healthy population.

## 2. Materials and methods

### 2.1. Breast cancer patients

The patients who were later defined as breast cancer group (BG) (n = 26) were recruited from the Hematology-Medical Oncology policlinic, Airlangga University Hospital, Surabaya, East Java, Indonesia, from among breast cancer outpatients between August 2019 and October 2019. The breast cancer diagnosis was confirmed by triple test approach. The inclusion criteria were as follows: female patients, more than 18 years old, early-stage breast cancer (Stage 1A, 1B, 2A, and 2B). The patients who had other malignancies were excluded from the study. Healthy subjects were recruited from age-matched and menstrual status-matched healthy volunteers. The entire healthy subject group has been proven to be healthy by physical examination. The healthy subjects were later defined as healthy group (HG). The subjects gave their written informed consent before participating in this study. This study has been reviewed for its ethical eligibility by Airlangga University Hospital Bioethical Committee, which was proven by Ethical Clearance Document No. 164/KEP/2019. Patient identity and data (age, sex, and menstrual status) were obtained from medical records and anamnesis.

### 2.2. Plasma preparation

Blood sample collection was performed in Hematology-Medical Oncology polyclinic, Airlangga University Hospital, Surabaya, East Java, Indonesia. Blood sample collection was performed after early-stage breast cancer diagnosis was confirmed and neoadjuvant chemotherapy procedure with mixed patient history of curative surgery. The blood sample was drawn from peripheral blood by venipuncture and needle aspiration of a 5-mL peripheral blood. The blood was collected into BD Vacutainer® EDTA tube (Becton, Dickinson and Company, Plymouth, UK). Plasma isolation was obtained by performing centrifugation of the 5-mL blood sample (1900 G; 10 min). The supernatant was collected without disturbing buffy-coat layer and recentrifuged (1900 G; 10 min) to obtain clear plasma. Blood plasma sample has advantages over serum sample materials, due to the presence of minimal hemolysis as a confounding variable [21].

### 2.3. RNA extraction

Total plasma RNA extraction was conducted using Qiagen miRNeasy serum/plasma kit (Cat: 217184, Lot: 163011798, Qiagen, Hilden, Germany) according to the protocol provided by kit manufacture. Total RNA content was measured using NanoDrop (Thermo Fisher Scientific Inc., Waltham, MA, USA).

### 2.4. cDNA (complementary DNA) synthesis 

Total isolated RNA from plasma was then transferred and underwent reverse transcription for producing cDNA. Reverse transcription was carried out using TaqMan MicroRNA reverse transcription kit (Cat.4366596, Lot 00783142, Applied Biosystems, Carlsbad, USA) by mixing 3 μl of RT primer and 5 μl RNA sample (1-10 ng RNA) with 7μl of reverse transcription reaction mix. The thermal cycle setting that used was 16 °C for 30 min, 42 °C for 30 min, and. 85 °C for 5 min. The synthesized cDNA was then stored at −20 °C before entering the quantitative real-time polymerase chain reaction (qRT-PCR). The primers used as endogenous control were Hsa-miR-21-3p (miRNA-21) and Hsa-miR-16-5p (miRNA-16). 

### 2.5. MiRNA-21 expression quantification

qRT-PCR from cDNA was performed using TaqMan MicroRNA assay kit (Applied Biosystems, California, USA) for miRNA-21, TaqMan endogenous control assay for miRNA-16 (Applied Biosystems, Waltham, MA, USA), and TaqMan Universal PCR master mix II, No UNG (Applied Biosystems, Waltham, USA) according to the protocol provided by respective manufacture. qRT-PCR started by mixing cDNA with PCR reaction mix. The thermal cycle setting for qRT-PCR was 95 °C for 10 min, 95 °C for 15 s (40 cycles), and 60 °C for 60 s. Sample banking and analysis were performed in Prodia Clinical Laboratory Jakarta. Relative miRNA-21 expression (2-ΔΔCt) was quantified through automatic quantification with miRNA-16 as an endogenous control. This simultaneous detection of miRNA-21 coupled with miRNA-16 as an endogenous control in the qRT-PCR improves the precision of miRNA-21 as a biomarker [21–23].

### 2.6. Data analysis

Data normality was assessed through Shapiro-Wilk test. Subject age was analyzed using independent t-test, and subjects’ menstrual status matching was assessed using Fisher’s exact test. MiRNA-21 expression comparison between HG and BG was analyzed using Mann-Whitney test. Analysis of miRNA-21 expression between HG and 1A, 1B, 2A, and 2B stage BG was performed utilizing Kruskall-Wallis test. Further analysis of miRNA-21 expression based on menstrual status separated for the entire subject and HG was performed using Mann-Whitney test, whereas independent t-test was used for assessing BG. MiRNA-21 cut-off point, sensitivity, and specificity were obtained through receiver operating characteristic (ROC) curve analysis. IBM SPSS Statistics v.24.0 (IBM Corp.) software was used for performing the entire statistical analysis. Statistical significance was set at the 0.05 probability level (P < 0.05).

## 3. Results

### 3.1. Subject characteristics

The mean ages of the HG and BG in this study were 49.750 ± 10.428 and 50.000 ± 10.987, respectively (P = 0.883). Menstrual status evaluation also showed an insignificant difference between both groups (P = 0.589). Indicated an age and menstrual status-matched group (Table 1).

**Table 1:** Subject characteristics.

Variable	Healthy control group(n = 16)	Breast cancer group(n = 26)	P
Age distribution (years)			
20-29	0	1	n.a
30-39	3	3	n.a
40-49	7	10	n.a
50-59	2	7	n.a
60-69	3	3	n.a
70-79	1	2	n.a
Age mean (years)	49.750 ± 10.428a	50.000 ± 10.987a	0.883b
Menstrual status			
Premenopause	56.25% (n = 9)	57.69% (n = 15)	0.589c
Menopause	43.75% (n = 7)	42.31% (n = 11)	
Breast cancer staging			
Stage 1A	-	15.38% (n=4)	n.a
Stage 2B	-	3.85% (n=1)	n.a
Stage 2A	-	34.62% (n=9)	n.a
Stage 2B	-	46.15% (n=12)	n.a

### 3.2. Circulating miRNA-21 expression comparison between HG and BG

**Figure 1:**
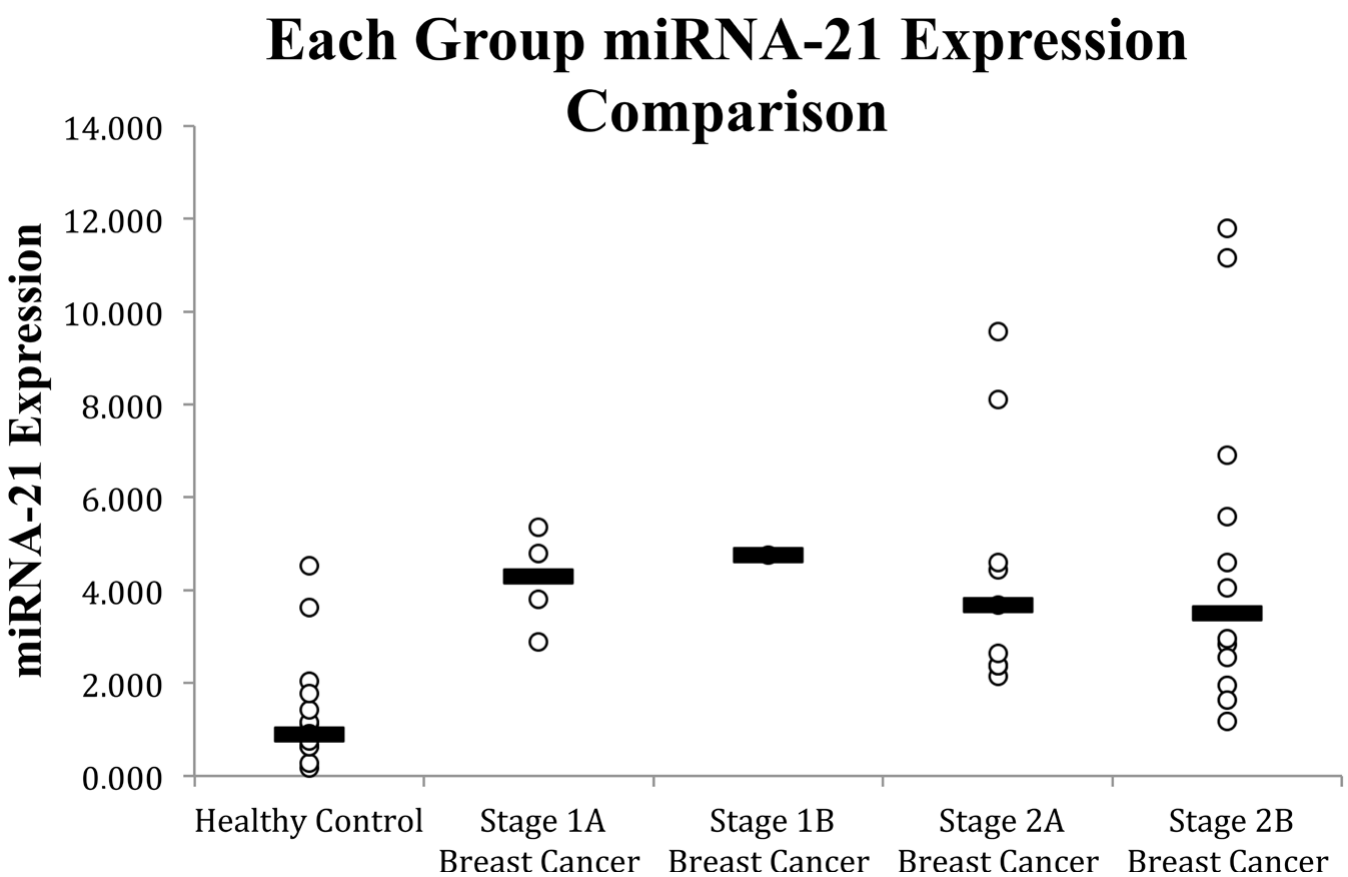
MiRNA-21 expression in 1A, 1B, 2A, and 2B stage breast cancer patients (BG) compared to healthy control subjects (HG). Note: White dots indicate circulating miRNA-21 expression data, black line indicates circulating miRNA-21 expression median.

There is a significant increase in miRNA-21 expression in breast cancer patients compared to healthy control subjects, indicated by the 4.364-fold increase in median miRNA-21 expression from HG compared to BG, 0.901 (0.164-4.364) and 3.933 (1.181-11.794), respectively (P = 0.001), based on Mann-Whitney test (Table 2). Evaluation of the miRNA-21 comparison in the specific breast cancer stage based on the Kruskall-Wallis test suggested that there is a significant difference in miRNA-21 expression across healthy control group (HG) and breast cancer subject group (stage 1A, 1B, 2A, and 2B BG) (P = 0.006) (Figure 1).

**Table 2:** Circulating miRNA-21 expression comparison between BG and HG.

Variable	Breast cancersubjects (n = 26)	Healthy control subjects (n = 16)	miRNA-21 expression change	P
miRNA-21 expression (2-ΔΔCt)	3.933(1.181-11.794)a	0.901(0.164-4.532)a	4.364times increase	0.001b

aThe results are described as median (min-max).bP-value is reported based on the analysis of Mann-Whitney U test (95% CI).

### 3.3. Circulating miRNA-21 expression based on menstrual status

Further analysis of miRNA-21 expression, in both HG and BG separately, revealed HG miRNA-21 expression of 1.123 (0.758 **-** 4.532) for premenopause subjects and 0.651 (0.164 **-** 0.414) for menopause subjects (0.578 times decrease, P = 0.031). On the contrary, in BG, we observed 1.720 times more miRNA-21 expression in premenopause subject (3.500 ± 1.517) and miRNA-21 expression of 6.021 ± 3.583 for the menopause subjects. However, miRNA-21 expression analysis on the entire subjects revealed an insignificant difference (P = 0.929) (Table 3).

**Table 3:** Circulating miRNA-21 expression comparison between premenopause and menopause subjects.

Variable	Groups	Premenopause	Menopause	miRNA-21expression change	P
miRNA-21 expression(2-ΔΔCt)	Healthy control subjects (n = 16)	1.123(0.758-4.532)a	0.651(0.164-0.414)a	0.578 times decrease	0.031c
	Breast cancersubjects (n = 26)	3.500 ± 1.517b	6.021 ± 3.583b	1.720 times increase	0.022d
	Total (n = 42)	2.594(0.758-6.916)a	2.912(0.164-11.794)a	1.123 times increase	0.929c

### 3.4. Circulating miRNA-21 expression in correlation with age

There was an insignificant correlation between subjects’ ages with miRNA-21 expression, whereas the correlation was measured in the entire subjects (P = 0.512), in HG (P = 0.166), or BG (P = 0.057).

### 3.5. Circulating miRNA-21 expression cut-off value, sensitivity, and specificity

**Figure 2:**
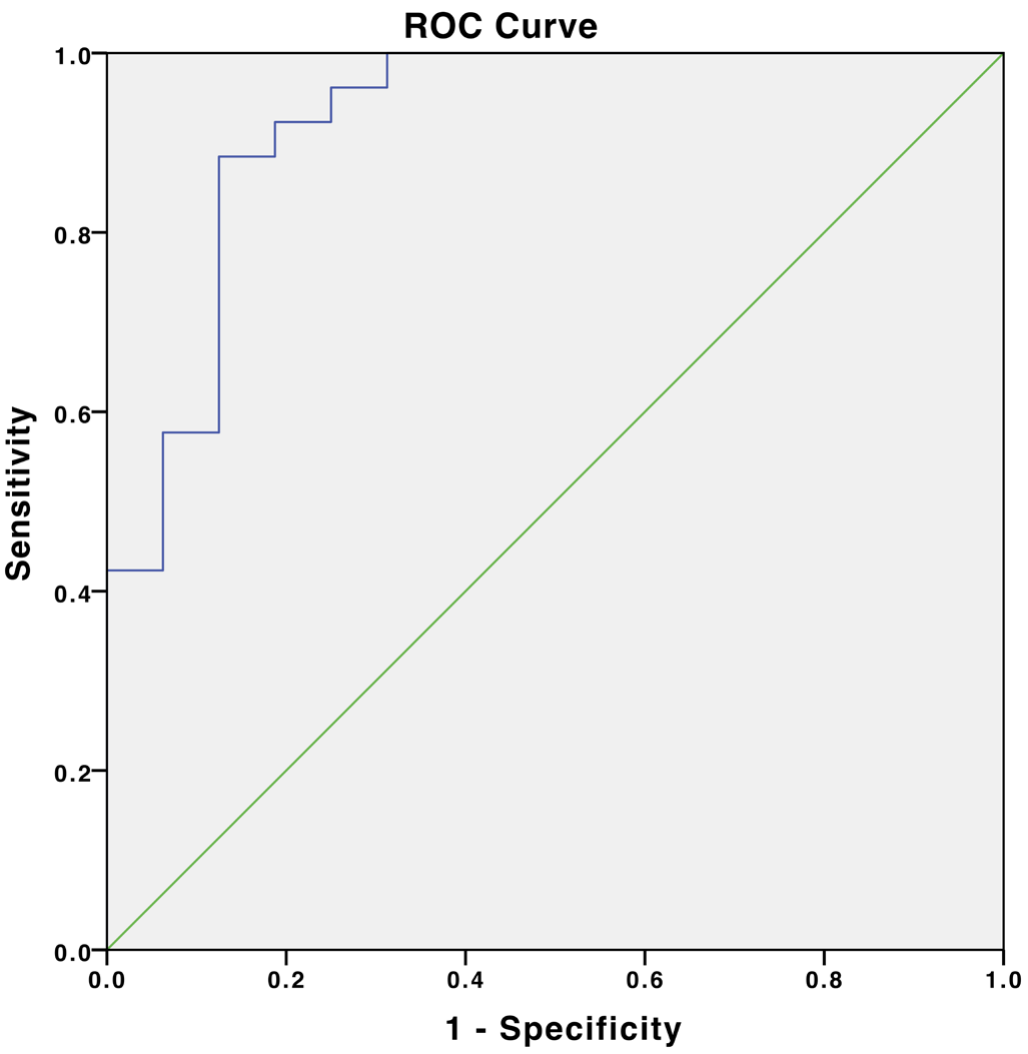
MiRNA-21 expression ROC curve.

The ROC (receiver operating characteristic) curve analysis in this study reveals circulating plasma miRNA-21 expression cut-off value as 1.86, with 92.3% area under the ROC curve (ROC-AUC) (95% confidence interval: 83.4%-100%). ROC curve evaluation yielded 92.3% sensitivity and 81.2% specificity (Figure 2).

## 4. Discussion

In our study, we observed that the mean age of HG was 50.000 ± 10.428 and BG was 50.000 ± 10.987 (P = 0.895). Also, the menstrual status in both groups showed an insignificant difference (P = 0.589). This result suggested that this study could represent a population of adult female patients. Therefore, further analysis of other parameters between both groups could be performed with a relatively minimalized bias [20]. 

In this study, the median value of miRNA-21 circulating plasma expression in the breast cancer group was 3.933 (1.181 - 11.794) while in the healthy control group it was 0.901 (0.164-4.532), which indicated that overall circulating miRNA-21 expression in the BG tends to be greater than HG with a median increase of 4.364 times. This finding is consistent with a previous study carried out by Gao et.al.. In their study, healthy group’s miRNA-21 expression was 9.1, and it was 30.82 for the breast cancer groups (3.39 folds increase) [8]. Another study also reported similar results of 3.05 HG mean circulating miRNA-21 expression and 6.88 for BG (2.25 times increase) [24]. The variability of miRNA-21 based on the previous study might be caused by the variability of detection kit that was used, patient demographic characteristics, and differences in the sample population, including genetic and epigenetic variances. The significant difference in miRNA-21 expression across HG and BG for each stage (stage 1A, 1B, 2A, and 2B) suggests that each group showed a significant difference; however, evaluation of a single group breast cancer progression could not be generalized as the sample does not tend to be evenly distributed, especially in the 1B stage breast cancer group which was composed only of 1 patient’s data. Hence, further evaluation for each group through post hoc analysis is required.

Additionally, we assess miRNA-21 expression difference between premenopause and menopause subjects through total subject analysis, HG, and BG analysis. There is an insignificant circulating miRNA-21 expression difference between premenopause and menopause women across the entire subjects. However, this result might be caused by a conflicting miRNA-21 expression tendency in HG and BG as our study reported. 

In BG circulating miRNA-21 tends to be higher in menopause subjects, which was in line with previous findings [25]. Separated analysis on HG revealed a significant decrease in circulating miRNA-21 expression in menopause women. This finding might indicate that circulating miRNA-21 expression tends to decrease after menopause in normal settings. However, in breast cancer cases due to the various circumstances, circulating miRNA-21 expression is increased and contributes to oncogenesis [10,26]. Hence, larger studies that objectively confirm our finding are still needed. Further 2-time point circulating miRNA-21 evaluation before and after menopause study should be performed for further understanding of its role in breast cancer occurrence and determining its usage in diagnostic. 

The menopausal and premenopausal miRNA-21 level difference could be caused by several mechanisms that are related to aging and hormonal metabolism changes. Menopause substantially changes women’s hormonal metabolism, especially in the metabolism of estrogen. Before menopause, estrogen production is mainly coordinated by hypothalamus-pituitary-ovarian axis with estradiol as the most prominent estrogen produced. However, after menopause, the estrogen main producer is switched to adipose tissues with estrone as the major estrogen hormone in postmenopausal women [27]. These metabolic changes could eventually provide a distinct organ response towards different estrogen dominancy stimuli. It is reported that estrone and estradiol trigger distinct transcriptome in breast cancer. Estrone exhibits NFκB-related inflammatory cytokines genes induction, whereas estradiol suppressed this mechanism in breast tissues. Moreover, in vivo study in the ER-positive breast cancer model showed that estrone promotes tumor progression, inflammation response, and increases ALDH1 activity as a breast cancer stem cell marker that suggests a worse breast cancer prognosis [28].

Furthermore, a distinctive signaling complex of miRNA-21 and NFκB in a specific type of cells was also identified. In epithelial cells, miRNA-21 downregulate PTEN, activate AKT, and upregulate NF-κB with a possibility to exist as a positive feedback loop as demonstrated in a nontumorigenic human mammary cell line (MCF-10A) and might be present in various breast cancer cells [29]. Nevertheless, as our study utilized circulating plasma miRNA-21 level, the balance between miRNA-21 and NF-κB signaling might be affected by other types of cells besides breast epithelial cells that possessed discrete miRNA-21 and NF-κB signaling cascades. This model might be explained by the opposite miRNA-21 and NF-κB interaction in the LPS-stimulated macrophage that exhibits PDCD4 downregulation, followed by NF-κB downregulation as a negative feedback loop [30]. Therefore, based on the previous findings, it is suggested that our findings of the lower miRNA-21 expression level in postmenopausal healthy subjects and its counterpart might be linked to miRNA-21 and NF-κB feedback loop balance in various cells without overlooking human body as a whole entity. Additionally, a further extent of miRNA-21 and NF-κB feedback loop balance should be performed for understanding a bigger picture of this phenomenon.

Evaluation of hormonal aspects should also be explored as menstrual status is connected with hormonal exposure. A previous study reported that the miRNA-21 tends to be correlated with the triple-negative breast cancer intrinsic subtype [26]. Yet, another study stated that there is an insignificant miRNA-21 expression difference in the absence and presence of progesterone receptor (PR), estrogen receptor (ER), and human epidermal growth factor receptor-2 (HER2) [25]. 

Correlation between miRNA-21 and age in total subject, HG, and BG showed no significant correlation. These results are in line with the prior study of miRNA-21 correlation [24]. An insignificant correlation between miRNA-21 level and age might indicate that circulating miRNA-21 expression is not related to aging mechanism. 

The ROC curve yields circulating plasma miRNA-21 expression cut-off value of 1.86 with an area under the ROC curve (ROC-AUC) of 92.3% (95% confidence interval: 83.4%-100%), the sensitivity of 92.3%, and specificity of 81.2%. These results are consistent with the previous study by Gao et.al. which reported 92.9% ROC - AUC, with a difference in overall sensitivity (82.6%), and overall specificity (87.3%). This study also states that miRNA-21 showed a higher sensitivity for detecting stage 1 breast cancer (92.24%) [8]. Another study by Wang and Zhang reported a lower ROC - AUC (88%), specificity (87.7%), and sensitivity (80%) with a higher 4.58 cut-off value [24]. A study by Mar-Aguilar et. al. also reported decent ROC-AUC (95%), specificity (80%), and sensitivity (94.40%), with 6.48 as a cut-off value [19]. The difference between the results of diagnostic values ​​with some previous studies in cut-off values, sensitivity, and specificity might occur due to the differences in the subjects’ genetic makeup, breast cancer stages, sample materials, RNA extraction methods, qRT-PCR analysis methods, and other confounding factors.

Limitation of this study includes the following: limited total subjects (the limitation was caused by subject recruitment timeframe from August 2019 to October 2019), uneven distribution of each breast cancer stage subject representation (1A, 1B, 2A, and 2B) with only one subject included in 1B group absence of breast cancer subtype data, absence of BG estrogen receptor status, absence of serum estrogen level data, hormonal-affecting medication data, contraception data, and mixed subject history of curative surgery. 

In conclusion, the expression of circulating plasma miRNA-21 in the early stage of breast cancer is increased compared to healthy control subjects. Therefore, suggesting that the detection of circulating miRNA-21 expression might serve as a potential diagnostic tool for detecting early-stage breast cancer. Moreover, based on the tendency of increasing circulating miRNA-21 expression in menopause breast cancer patients compared to premenopause breast cancer patients with a contrary finding in HG, it is suggested that miRNA-21 might serve as a breast cancer risk predictor.

## Informed consent

The subjects have given their written informed consent before participating in this study. This study has been reviewed for its ethical eligibility by Airlangga University Hospital Bioethical Committee, proven by Ethical Clearance Document No. 164/KEP/2019.
